# Regionalization of Soil and Water Conservation Aimed at Ecosystem Services Improvement

**DOI:** 10.1038/s41598-020-60100-8

**Published:** 2020-02-26

**Authors:** Xiaoqian Hu, Zhongwu Li, Xiaodong Nie, Danyang Wang, Jinquan Huang, Chuxiong Deng, Lin Shi, Lingxia Wang, Ke Ning

**Affiliations:** 1 College of Resources and Environmental Sciences, Hunan Normal University, Changsha, 410081 China; 2 College of Environmental Science and Engineering, Hunan University, Changsha, 410082 China; 3 Hunan Water Resources and Hydropower Research Institute, Changsha, 410007 China; 4 Yangtze River Scientific Research Institute, Wuhan, 430010 China

**Keywords:** Ecology, Environmental sciences, Natural hazards

## Abstract

To effectively control soil erosion, three hierarchies of the National Soil and Water Conservation Regionalization Scheme have been established in China. However, the scheme has its limits, which can be summarized by two points: first, the tertiary hierarchy functional region exhibits obvious heterogeneity; second, the ecosystem function does not influence the regionalization scheme results during the process of regionalization. To enhance the guidance of the regionalization, a new indicator system included soil erosion risk, soil erosion intensity and ecosystem service value was developed to explore the subdivision of the tertiary hierarchy functional region. Moreover a scheme for the subdivision of the tertiary hierarchy functional region was formed. In this scheme, the central Hunan hilly soil conservation and living environmental protection section was divided into three subregions: Luoxiao-Xuefeng Mountain high ecological value section, Xiangjiang middle and downstream medium ecological value section, and Hengyang Basin low ecological value section. Specifically, with regard to soil and water conservation regionalization, the concept of subregions within the tertiary hierarchy-based functional region was proposed and the new indicator system that highlighted ecosystem functions was applied for the first time on a regional scales; this method provides a new way of thinking about other regionalization schemes.

## Introduction

Soil and water loss has been regarded as one of the most global environmental problems, affecting the ecosystem and the safety of lives and property^1^. The prevention of soil erosion and improvement to the eco-environment are required for sustainable development^2^. The regionalization of soil and water conservation is based on the analysis of regional physiographical conditions, eco-environmental statuses, characteristics of soil erosion and regional development conditions, which are used to determine the direction of soil and water conservation planning and approaches to prevent soil erosion^3^. This analysis also benefits the reasonable distribution of soil conservation measures and is of great significance to promote the rational utilization of regional soil resources and the development of society, economy and the environment. Therefore, the regionalization of soil and water conservation is of great practical significance for the construction of an ecological civilization.

Regionalization is the foundation of planning for soil and water conservation and the precondition for the rational layout of soil and water conservation measures^4^. Studies on the methods of regionalization for soil erosion control are of practical significance. Regionalization of soil and water conservation in China has a history of nearly 70 years. The regionalization methods have been constantly improved from qualitative to quantitative. The indicator system for regionalization has also been improved. Before 1990, the regionalization of soil and water conservation was mainly based on practical work experience. For example, Xu *et al*.^5^ divided Hui’an County into three soil and water conservation areas based on experience. Recently, mathematical methods have been applied to the regionalization of soil and water conservation, such as fuzzy clustering and Bayesian stepwise discrimination. With the development of the “3 S” technology, i.e., remote sensing (RS), geography information systems (GIS), and global positioning systems (GPS), researchers have combined mathematical methods with RS and GIS for regionalization^3^. The combination of mathematical methods and “3 S” technology was able to achieve a quantitative regionalization process, and visualization of regionalization. For example, Zhang *et al*.^6^ divided Shanxi Province into four first-level districts and eight second-level districts by using a combination of statistical analysis and spatial overlay analysis. The development of indicator systems reflects the expansion of the soil and water conservation regionalization concept. First, the indicator systems considered only soil erosion types. After that, factors such as natural conditions and socioeconomics were gradually integrated into the indicator system. The current indicator system for the regionalization of soil and water conservation generally consists of natural, socioeconomic, land use and soil erosion factors. For example, when Chu *et al*.^7^ carried out regionalization of soil and water conservation in Liaoning Province based on the multiple tests method, 21 indicators were selected and divided into four element layers. When Mi *et al*.^8^ studied the regionalization of soil and water conservation in Yunnan Province, they selected 86 indicators including natural, socioeconomic, land use and soil erosion indicators, to construct an index system. Wang *et al*.^4^ quantified the dispersion degrees of the indicators for large-scale, regional, provincial and county scale soil and water conservation divisions, which were 0.80, 0.64, 0.90, and 0.62, respectively. A higher the degree of the dispersion indicates a greater the difference in the selection of indicators. Therefore, the current indicator system suffers from the problem of blindly loading indicators into the regionalization indicator system.

Water and soil are the most precious resources for human beings. Soil erosion is a concentrated reflection of land degradation and loss of ecosystem function and is directly related to national ecological security. The functional definition of soil and water conservation ecological services refers to the comprehensive effect of the use of various measures adopted during the soil and water conservation process on maintaining, improving and protecting natural environmental conditions^9^. For example, the “Grain-for-Green” program implemented by China in 1999 caused large-scale land-use changes^10^, thus affecting the ecosystem services (e.g., water conservation, soil conservation, carbon sequestration and oxygen release, etc.)^10–12^. Moreover, it has been a trend to combine soil and water conservation regionalization with the ecosystem service function in recent years. For example, the “National Soil and Water Conservation Regionalization Scheme (Trial)” is currently used in China. The indicator system of this regionalization mainly considers the topographic features, hydrothermal conditions, and hydrology, soil, land use, and socioeconomic indicators. Based on this indicator system, China (excluding the Hong Kong, Macao and Taiwan regions) was divided into eight regions, 41 subregions and 117 sections according to a three-tier hierarchy^2^. After that, the soil and water conservation functions of each three-tier area were evaluated to determine the leading basic functions of each area, and the same names were used to reflect the functions. The dominant soil and water conservation function was reflected by the name. The three-tier hierarchy names were established in the form of “geographical location + landform + leading functions of soil and water conservation”. This method is mostly used in research on the current regionalization of soil and water conservation^7,8,13,14^. Therefore, this combination is reflected in only the identification of the leading function after regionalization, and the current indicator system does not highlight the impact of ecosystem services on the regionalization process^4^.

The soil and water conservation regionalization scheme, which is currently used, has identified the direction of soil and water conservation planning, and the introduction of the scheme has caused an upsurge of the regionalization of soil and water conservation at different scales, such as provinces, cities, counties and river basins^15^. However, the section of this scheme suffers from the problem of having areas that are too large. When the area of the divided region is very large, and regional differences in soil erosion driving factors are significant, the guidance for the layout of soil and water conservation measures will be poor^6^. Thus, to enhance the guidance of the soil and water conservation regionalization scheme, it is necessary to refine these tertiary regions. The central Hunan hilly soil conservation and living environmental protection section is an important grain production base for China. However, this area suffers from serious soil erosion. The regional heterogeneity is significant in this area, which is not conducive to the formulation of soil resource protection policies by local governments. Therefore, focusing on the problems that soil and water conservation measures cannot be adapted to local conditions and that ecosystem services have not been highlighted in the soil and water conservation regionalization indicator system, this paper selects the central Hunan hilly soil conservation and living environmental protection section as a case to discuss the subdivision of the third-level subregion. Based on GIS technology, this paper constructed a soil and water conservation regionalization indicator system for the tertiary subregion to enhance ecosystem service functions and this system was used to further divide the central Hunan section. The results of this research are of great significance for improving soil and water conservation regionalization and can be a reference for formulating soil conservation policies.

## Materials and Methods

### Study area

**Figure 1:**
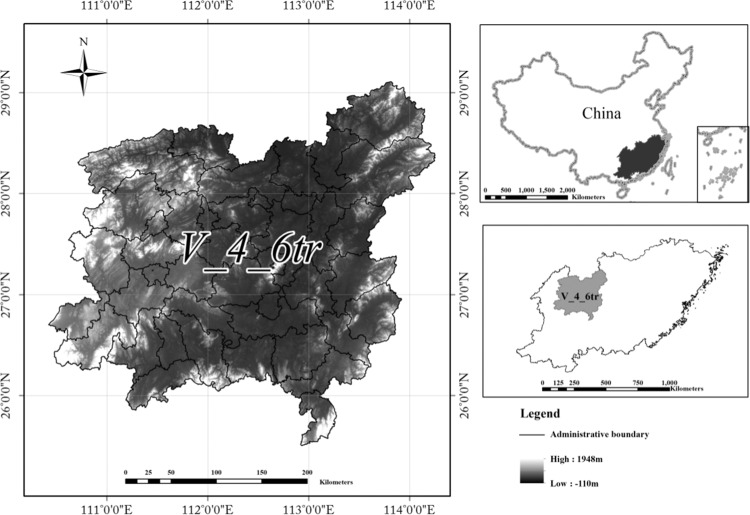
Location Map of the Central Hunan hilly soil conservation and living environmental protection section. Map generated using the ArcGIS 10.5 software (ESRI Inc., California, USA. URL: http://www.esri.com/software/arcgis/arcgis-for-desktop). (Source of data: Scheme of soil and water conservation regionalization in China).

The study area is the central Hunan hilly soil conservation and living environmental protection section (V-4-6-tr), which is one of the 117 sections in the soil and water conservation regionalization scheme, and belongs to the secondary regionalization south Yangtze River hilly subregion (V-4), with the primary regionalization being the South China red soil region (V). The study area is approximately 86,453 km^2^ and contains 58 administrative countries (Fig. 1). The tertiary hierarchy functional region has a subtropical humid monsoon climate. The annual precipitation is 1217–2252  mm. The Anhua and Taojiang counties are the rainstorm centers, and the precipitation is over 1600 mm, while the precipitation is low in the Hengyang Basin at approximately 1200  mm. The terrain is high in the east, west and south, with the Luoxiao Mountains and Xuefeng Mountains. The center of this area is hills and plains. The relative height is approxomately 2000 m. The central part of this area is covered by fertile plain, abundant precipitation, extensive solar energy, and vast territory, which is suitable for the agricultural development and is the reason for the high agricultural commodity rate in the area, and the economy is developed. This tertiary hierarchy functional region is one of the areas that suffers suffer severe soil erosion in the South China red soil region. At present, the area of soil erosion is 157217.10 km^2^, accounting for 17.6% of this tertiary hierarchy functional region. The study area is dominated by both light and moderate soil erosion, covering approximately 72.54% and 19.88%, respectively.

### Data source

The data used in this research mainly cover geomorphological and socioeconomic aspects, land use and soil loss. Geomorphological data were acquired from a digital elevation model (DEM) by GIS, and the resolution of the DEM was 30  m. The socioeconomic data were obtained from the statistical yearbook of Hunan Province and government websites of various counties. This paper used the average value of ten years from 2006 to 2016 for analysis. Land-use type data were mainly interpreted by a human-computer interactive visual interpretation based on the Landsat-8 land use/cover thematic data from 2015.

### Regionalization method

Based on previous studies and aimed at the shortcomings of the current indicator system for soil and water conservation regionalization, this paper constructed a new indicator system composed of soil erosion risk, soil erosion intensity and ecosystem service function and included 19 factors. In addition to the correlation with soil erosion, the relative independence of each index and the representativeness of the index were also considered^16^. The risk of soil erosion reflects the combined influence of nature and humans. The landform is the internal factor that leads to soil erosion.

The complexity of terrain and the diversity of climate lead to differences in soil erosion risk between different regions^17^. Surface fluctuations, gully density, annual average rainfall and vegetation coverage rate were selected to reflect the differences in the natural environment of the region. Surface fluctuation refers to the distance between the highest and lowest elevations in a region. The terrain of the research area is high on the periphery and low in the middle region, and the relative altitude is approximately 1,964  m. The large topographic relief promotes soil erosion. Gully density is closely related to regional topography, slope, precipitation, runoff characteristics, soil erosion resistance, etc., and can be a measurement of the degree of surface fragmentation; these characteristic are of great importance to soil erosion monitoring and planning^18^. Rainfall is an important inducing factor of soil erosion^19^. Vegetation can buffer the scour of rainfall and enhance the stability of surface materials. The effect of development on soil erosion is bidirectional. On the one hand, development promotes soil erosion; on the other hand, it serves as the economic foundation for soil and water conservation efforts. This paper reflects the difference in regional development by using population density, urbanization rate and per capita GDP. At the same time, the change in land use type also leads to changes in natural phenomena and ecological processes^20^, directly affecting the ecosystem and the well-being of the lifelong resident^21^. The percentages of cultivated and construction lands were selected to reflect the impacts of land use type. The differences in landform and soil and water conservation abilities lead to differential risks of soil erosion is different in different regions.

Soil erosion has led to a plethora of environmental problems. For example, the soil layer has become thin, soil nutrients have decreased, and the adaptability of land use has been altered^22^. The intensity of soil erosion reflects the status of regional soil erosion, which has a great influence on determining the personnel and funding investments the distribution of soil and water conservation measures. This factor reflects the current situation of soil erosion in different evaluation units by the ratio of five different erosion grades: light, moderate, intense, extremely intense, and drastic. The classification of soil erosion grades is based on China’s current classification standards, that is, the “standards for classification and gradation of soil erosion” (SL 190–2007) issued by the People’s Republic of China Water Conservancy Industry Standard^23^.

Ecosystem services ensure that people obtain benefits directly or indirectly from the ecosystem^14,20,22,24^. Ecosystem service functions are vital for human survival and are formed by ecosystems and ecological processes. On this basis and aimed at the problems of soil and water conservation, some researchers have put forward the concept of soil and water conservation ecosystem service functions which include water conservation, soil conservation, biodiversity maintenance, carbon sequestration, air purification, and human settlement environment maintenance^25,26^. Soil erosion is a collective reflection of ecological degradation. Ecosystems have self-recovery ability, which are related to the species diversity, structure and function of the ecosystem^27,28^. The degradation of ecosystems undermines ecosystem functioning and resilience, thus threatening the ability of the ecosystem to provide ecosystem services^29^, such as regulating the atmosphere, maintaining biodiversity, and maintaining soil fertility^30^. Ecosystem service values reflect the goods and services that ecosystems provide to humans^24^. Thus, to a certain extent, the value of ecosystem services can reflect the self-repair capacity of the ecosystem. The assessment of the value of ecosystem services is an important tool to encourage policy makers to pay more attention to the ecosystem. Therefore, taking the value of the ecosystem service function as a factor of regionalization, it is necessary to consider the value of ecosystem service functions in zoning. In this paper, the total value of ecosystem services in the central Hunan hilly soil conservation and living environmental protection section is evaluated by five factors: soil conservation, biodiversity maintenance, nutrient cycling maintenance, environmental purification, and hydrological regulation. At present, there are many methods to assess the value of ecosystem services. The equivalent factor method proposed by Xie can achieve the rapid valuation of ecosystem services and has been used by many scholars to assess the value of ecosystem services^31–34^. The equivalent factor of the standard unit system refers to the economic value of food produced by a hectare of farmland, then this value is taken as a reference, and is combined with expert knowledge to determine the equivalent factors for other ecosystem services^35–37^. This method is suitable for the study region and avoids problems such as difficulties in data collection.

Then, the main factors affecting the prevention and control of soil erosion in this area are screened according to local environmental conditions. Finally, an indicator system for the subregion of tertiary soil and water conservation regionalization is constructed (Table 1).

**Table 1:** The indicator system used for sub-division of center Hunan hilly soil conservation and living environmental protection section.

Target	Elements	Index	weigh
Central Hunan hilly soil conservation and living environmental protection section soil and water conservation regionalization	soil erosion risk	surface fluctuation	0.10
		gully density	0.11
		perennial average rainfall	0.22
		vegetation coverage	0.08
		population density	0.07
		urbanization rate	0.14
		GDP per capita	0.10
		cultivated land ratio	0.11
		construction land ratio	0.07
	soil erosion intensity	light erosion area ratio	0.14
		moderate erosion area ratio	0.13
		intensity erosion area ratio	0.12
		extreme intensity erosion area ratio	0.31
		drastic erosion area ratio	0.30
	ecosystem service value	purify the environment	0.20
		soil conservation	0.21
		nutrient maintain	0.21
		biodiversity conservation	0.20
		hydrological adjusting	0.18

The entropy method is an objective weighting method. Entropy is a measure of uncertainty and is consistent with uncertainty. Entropy can be used to determine the degree of dispersion of an index. If the degree of dispersion is large, the weight of the index will be correspondingly large. This method has been widely used in model evaluation^38^. The entropy method is used to determine the weight of each index in this paper (Table 1). Then, we can obtain the values of three elements.

The soil erosion risk is calculated as:

1 $${I}_{e}=\mathop{\sum }\limits_{i=1}^{N}{W}_{i}{P}_{i}$$

where *I*_*e*_ represents the index of soil erosion risk. *N* is the number of factors. *P*_*i*_ and *W*_*i*_ represent the value and weight of the index, respectively.

The Chinese soil loss equation (CSLE)^39^ was used to calculate the soil loss in the study area in 2015, as this equation is suited to the Chinese national condition, and the model can reflect the impact of soil and water conservation measures on soil erosion. The CSLE model can be expressed as follows:

2 $${\rm{M}}={\rm{R}}\times {\rm{K}}\times {\rm{LS}}\times {\rm{B}}\times {\rm{E}}\times {\rm{T}}$$

where *M* is the average soil loss per unit area by erosion (t hm^−2^ a^−1^), *R* is the rainfall erosivity factor (MJ mm hm^−2^ h^−1^ a^−1^), *K* is the soil erodibility factor ((t hm^2^ h hm^−2^ MJ^−1^ mm^−1^), *LS* is the slope length and slope factor (dimensionless), *B* is the biological measure factor (dimensionless), *E* is the engineering measure factor (dimensionless), and *T* is the cultivation measures factor (dimensionless).

The rainfall erosivity factor is calculated based on measured data from 298 weather stations^40^ (Eq. 3). Soil erodibility factors are calculated using the erosion-productivity impact calculator (EPIC) model^41^ based on data from the second soil census in China (Eq. 4). The slope and slope length factors are calculated using the formula of the general soil loss equation^42^ (Eq. 5).The CSLE contains three soil and water conservation measures factors^43^. The biological measure factor is based on land use data and vegetation cover data (Table 2). The engineering measures factor is based on the data of the soil and water conservation bulletins in China’s provinces. The engineering measures we mainly considered in this paper are terraces (Eq. 6). The factors of farming measures are obtained by using the data from the Chinese agricultural zoning map and the soil and the assignment data of the water conservation engineering measures factor.

3 $${\rm{R}}=\mathop{\sum }\limits_{i=1}^{12}(\,-\,1.5527+0.1792{P}_{i})$$

4 $$\begin{array}{c}{\rm{K}}=0.1317\{0.2+0.3exp\,[-0.0256SAN(1-\frac{SIL}{100})]\}\ast {(\frac{SIL}{CLA-SIL})}^{0.3}\\ \ast (1-\frac{0.25C}{C+\exp (3.72-0.95C)})\ast (1-\frac{0.7SNI}{SNI+\exp (-5.51+22.9SNI)})\end{array}$$

5 $${\rm{LS}}={(\frac{L}{72.6})}^{m}\ast (65.41\,\sin \,\theta +4.56\,\sin \,\theta +0.065)$$

6 $${\rm{E}}=\frac{[({S}_{T}\ast {E}_{T})+({S}_{G}-{S}_{T})]}{{S}_{G}}$$

where P_i_ is the rainfall in month i. SAN, SIL, CLA and C are the contents of sand, silt, clay and organic carbon, respectively. The sizes of SAN, SIL and CLA are 0.050–2.00 mm, 0.002–0.050 mm, and <0.002  mm, respectively. SNI = −SN/100. L is the slope length. θ is the slope, and m is the index of slope length (Eq. 9), which varies with the slope (Eq. 6). T is the terrace. G is the tillage measure.

7 $${\rm{m}}=\{\begin{array}{r}0.2,\theta  < 0.57^\circ \\ 0.3,0.57^\circ \le \theta  < 1.72^\circ \\ 0.4,\,1.72^\circ \le \theta  < 2.86^\circ \\ 0.5,2.86^\circ \le \theta \end{array}$$

**Table 2:** The value of biological measure factor.

landuse	vegetation coverage (%)	B	landuse	vegetation coverage (%)	B
woodlands	0–20	0.100	grassland	0–20	0.450
	20–40	0.080		20–40	0.240
	40–60	0.060		40–60	0.150
	60–80	0.020		60–80	0.090
	80–100	0.004		80–100	0.043
Waters	—	0.000	flat ground	—	0.230
construction land	—	0.353	slope cropland	—	0.470

The classification of soil erosion intensity is divided according to the soil erosion intensity grading standard (SL19-2007) based on the soil loss per unit area every year. Then the soil erosion intensity index can be calculated as:

8 $${\rm{E}}=\mathop{\sum }\limits_{i=1}^{N}{{C}_{i}}^{{A}_{i}}/S$$

where *E* represents the comprehensive index of soil erosion; *N* is the number of factors; *C*_*i*_ represents the weight of erosion intensity; *A*_*i*_ represents the area of soil erosion in the evaluation unit; and *S* represents the total area of the study region.

The ecosystem service value is calculated as:

9 $${\rm{S}}=\mathop{\sum }\limits_{i=1}^{N}{W}_{i}{P}_{i}$$

where, *S* represents the ecosystem service functional value; *N* is the number of factors; *W*_*i*_ and *P*_*i*_ represent the weight and value of ecosystem functions. The calculation of ecosystem services value is based on the combination of land use data and the ecosystem services value equivalent factor table^36^.

**Figure 2:**
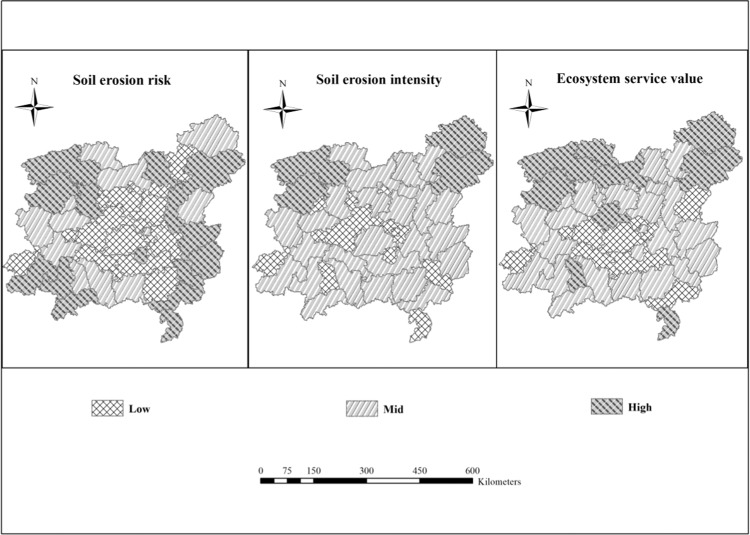
Classification results of three elements. Map generated using the ArcGIS 10.5 software (ESRI Inc., California, USA. URL: http://www.esri.com/software/arcgis/arcgis-for-desktop).

**Figure 3:**
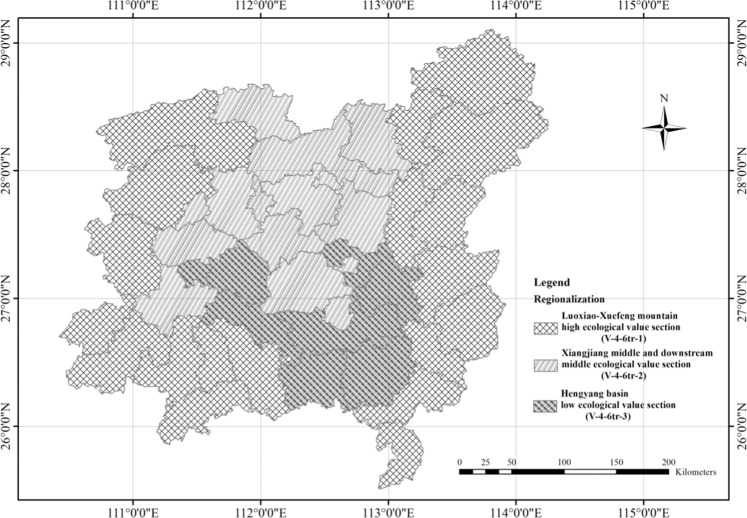
Regionalization Map of soil and water conservation in central Hunan hilly soil conservation and living environmental protection section. Map generated using the ArcGIS 10.5 software (ESRI Inc., California, USA. URL: http://www.esri.com/software/arcgis/arcgis-for-desktop).

At present, the combination of the analytic hierarchy process (AHP) method and layer overlay analysis is widely used for the determination of weights and regionalization^3,44,45^. The AHP method is a widely used method to calculate the weights of synthetic assessment indexes^16^, and it is a combined qualitative and quantitative hierarchical weight analysis method^46^. This paper used the AHP method to determine the weight of soil erosion risk, soil erosion intensity and the ecosystem service function (Table 3). The layer overlay analysis method can obtain new properties and relationships by stacking multiple layers of spatial data. The results of the regionalization obtained by this method are intuitive and reliable. First, with the support of GIS, we drew the distribution maps of the three elements, namely, soil erosion risk, soil erosion intensity and ecosystem service value (Fig. 2). Then, based on the spatial overlay technology of GIS, an arithmetic expression was constructed based on the weights of the elements obtained by the AHP method and was used to create the conditional layer overlay for each element layer. With the reclassification in GIS, the preliminary regionalization map of the center Hunan hilly soil conservation and living environmental protection section was obtained. The principle of the natural breaks method based on the principle of minimal intragroup differences and maximum intergroup differences was used to grade^47^. Finally, the quantitative regionalization results were adjusted appropriately according to the actual regional development planning and expert opinion. This paper obtained the schemes for subregions of tertiary soil and water conservation regionalization with the new indicator system (Fig. 3).

**Table 3:** Analytic hierarchy process calculates matrix and weight.

soil and water regionalization	soil erosion risk	soil erosion intensity	ecosystem service functions	weight
soil erosion risk	1	2	1/2	0.3
soil erosion intensity	1/2	1	1/2	0.2
ecosystem service value	2	2	1	0.5

## Results and Discussion

Based on the modified indicator system and supported by the spatial analysis function of ArcMap, the central Hunan hilly soil conservation and living environmental protection section was further divided into three subregions: Luoxiao-Xuefeng mountain high ecological value section (V-4-6tr-1), Xiangjiang middle and downstream middle ecological value section (V-4-6tr-2), and Hengyang Basin low ecological value section (V-4-6tr-3). The naming for the regionalization units of soil and water conservation was based on the expression of the regionalization results. The nomenclature for the soil and water conservation regionalization was mainly based on the concise principle characteristics and reflected the characteristics of the geographical location of the area. The naming method used in this study is “regional geographic location + ecosystem service value”.

The Luoxiao and Xuefeng Mountains are located in central Hunan Province with a total area of 48,394 km^2^. Moreover, this region is an important ecological barrier area in Hunan Province. The dominant regional geomorphology is mountains, most of which reach 1000  m. This province has abundant rainfall and frequent rainstorms, while the steep slope accelerates landslides and gully erosion, resulting in high soil erosion risk^48^. Furthermore, the Luoxiao Mountain is rich in mineral resources^49^ and is a key development zone and unreasonable development has aggravated soil erosion in this area. Nevertheless, there are rich forest resources, with forestland accounting for 47.69% of the total area of the region. Forest ecosystems can mitigate the impact of precipitation, and the diversity of forest ecosystems is conducive to the self-recovery and regulation of the ecosystem^50,51^. Xuefeng Mountain is a key ecological function area for Hunan Province. The ecosystem service value density is 3–4 billion $/km^2^. The hydrological adjustment and biodiversity functions of this area were significantly better than those of the other subregions. The self-restoring capacity is strong, and the soil erosion prevention and supervision should be strengthened in this region, such as via the control of mineral resource exploitation. Protecting the natural ecological zone around the Luoxiao and Xuefeng Mountains is vital for vegetation restoration, water quality maintenance, and the construction of livable environment constructions.

The Xiangjiang middle and downstream middle ecological value section is mainly located in the middle and downstream areas of the Xiangjiang River, with a total area of 23,707 km^2^. This section is dominated by light and moderate soil erosion and mainly occurs in steep-slope farmland and sparse vegetation. It is necessary to strengthen the protection of vegetation and focus on the improvement of steep-slope farmland. The northern part of this section is an integral development zone where the control of development and construction needs to be strengthened. The south is the main area of agricultural production in China, with fertile land and a high land utilization rate. When environmental protection projects are closely related to benefits for the residents, the enthusiasm of residents can be greatly increased^52^. Therefore, the development of economic agriculture based on soil and water conservation should be vigorously promoted to ensure the benefits for residents while promoting ecological protection^53^; this process would achieve the mutual success of both economic development and environmental protection. Only in this way can the goal of sustainable development be truly realized. To control soil erosion, local vegetation in sparse forestland should be planted to diversify its structure and function and enhance ecological diversity^54,55^. In addition, aquatic ecosystems have been developed in this region. The risk of soil erosion in this zone is above medium level, and the density of the ecosystem service function value in each county is 2–3 billion $/km^2^, which is slightly lower than that in the Luoxiao-Xuefeng mountain zone. Based on strengthening prevention, protection and natural restoration, human disturbances, such as supplemental engineering measures, should be appropriately increased in this region to improve the living environment.

The Hengyang Basin low ecological value section is located in the middle of Xiangjiang and has a mainly hilly topography. The study area is approximately 14,082 km^2^, and the population density is 446.6 people/km^2^. This region is also one of the major agricultural areas in China^56^, with agricultural acreage accounting for 40.8% of the total area. Soil erosion is serious over the steep-slope farmland, economic forests, and areas of sparse vegetation. Despite the low level of soil erosion risk, and the light to moderate level of soil erosion degree, this area has poor environment and low ecosystem service value. This is the key treatment region of central Hunan hilly soil conservation and living environmental protection section. An underdeveloped economy leads to environmental degradation, which in turn exacerbates poverty^37,52^. For regions with serious soil erosion and ecosystem destruction, vegetation rejuvenation should be carried out^3,57,58^. The preconditions for optimizing the eco-agricultural environment and improving the human settlement environment include building economic forests with both economic and ecological value, rectifying steep-slope farmland, improving agricultural technology, and improving supporting infrastructure for agricultural development. For example, when soil quality is poor, soil fertility can be improved through soil modifiers, such as biomass, which improves crop productivity^59–62^. Biological, engineering and agricultural technical measures should be combined to control soil erosion and reduce the vulnerability to soil erosion^63^, improve the stability of the ecosystem and promote the harmonious development between humans and nature.

This research filters the indexes by theoretical analysis and frequency statistics. Then 19 indexes are selected for the regionalization index, referring to expert opinions. In the process of regionalization, we enhance the impact of ecosystem services by their weights. Compared to the distribution of the main functional areas and strategic patterns of ecological security in Hunan Province, China, the results are in good agreement. In addition, the results of the regionalization are also consistent with the topography. Hence, the index we constructed is suitable for the central Hunan hilly soil conservation and living environmental protection section. This indicator system is only applicable to areas with similar characteristics to the study area because it is constructed based on the characteristics of the study area, including the geographical features, socioeconomic conditions and environmental status. For other regions that are significantly different from the study area, the methods used in this paper can be used to build an appropriate indicator system and obtain a regionalization scheme.

In addition, although the regionalization scheme in this study provides support for the focus of regional soil and water conservation work, it still has certain limitations in guiding the layout of soil and water conservation measures. For example, in regions with low ecosystem service value and low risk of soil erosion, measures can be deployed that have both ecological and economic functions, However, the layout of specific measures is still unclear and can be based on only practical experience.

## Conclusions

The tertiary hierarchy functional region offers guidance for the rational arrangement of soil and water conservation measures. The subdivision of the tertiary hierarchy functional region can enhance its efficiency. Here, we proposed an indicator system for the regionalization of soil and water conservation using the subdivision of tertiary regionalization, including soil erosion risk, soil erosion intensity and the ecosystem service function. Based on this indicator system, the central Hunan hilly soil conservation and living environmental protection section was divided into three subregions. The subregion of the tertiary soil and water conservation regionalization greatly weakened the regional heterogeneity and enhanced the practicality of the soil and water conservation regionalization scheme in China. In addition, the introduction of ecosystem services to the indicator system provided a new way of thinking for other regionalization techniques.

The combination of soil and water conservation planning and ecosystem services is the aim of future studies. The scheme for creating subregions of the tertiary hierarchy functional region oriented to the improvement of ecosystem services better handles the relationship between soil and water conservation, ecological environment, and socioeconomic development. This scheme provides support for determining the development direction of various types of areas (such as adjustment of land use, and industrial structure, etc.) and soil and water conservation control measures and provided scientific guidance for the configuration of the main control measures. The subregions of the tertiary hierarchy functional region can effectively guide the design of provincial soil and water conservation plans within a certain period. In future research, to provide more constructive guidance for soil and water conservation practices, simulations scenarios will be combined to simulate the layout of soil and water conservation measures by assessing their economic and ecological benefits to obtain the optimal layout plans for soil and water conservation measures.

## Data Availability

The datasets generated during and/or analyzed during the current study are available from the corresponding author on reasonable request.
